# A Russian Adaptation of the Emotional Contagion Scale

**DOI:** 10.3389/fpsyg.2022.872718

**Published:** 2022-08-17

**Authors:** Vladimir Kosonogov, Olga Kuskova

**Affiliations:** Institute for Cognitive Neurosciences, HSE University, Moscow, Russia

**Keywords:** emotional contagion, questionnaire, psychometrics, adaptation, empathy

## Abstract

The aim of the work was to develop and test the Russian version of the Emotional Contagion Scale. A sample of 518 volunteers from the general population filled in this questionnaire. We examined the one-factor model (all the items), the two-factor model (positive/negative), and the five-factor model (love/happiness/fear/anger/sadness). To measure its construct validity, we asked different subsamples to complete questionnaires of empathy and sensation seeking. The coefficients of test–retest reliability, internal consistency, and validity were acceptable. Only the one-factor model showed acceptable properties by all psychometric criteria. We also observed the gender effect, that is women were more contagious, according to the total scale and all subscales.

## Introduction

It was long established that emotions could be contagious: if someone is smiling in communication, it is likely for others to start smiling as well; and if someone is visibly unhappy, others could experience similar emotions. The phenomenon of emotional contagion implies the ability to perceive emotions in others and to “catch” those emotions by responding to emotional expressions (Hatfield et al., 1992, 1994). Research shows that emotional contagion happens on the level of facial expressions (Wild et al., 2001), body language, posture (Tia et al., 2011), and vocal expressions (Hietanen et al., 1998). Emotions can be spread not only in direct face-to-face communication but also *via* photos, video, and audio recordings (Lundqvist and Dimberg, 1995; Neumann and Strack, 2000; Wild et al., 2001; Papousek et al., 2009).

This makes the phenomenon of emotional contagion an important part of research in psychology, psychiatry, communication, commerce, and other fields related to the study of social interaction, affect, and social influence. Thus, thanks to contagion, psychotherapists can understand emotional states of their clients (Santos, 2015). However, at the same time, they should regulate their own emotions in order to stay independent from them (Rogers, 1975). Contagion has also been shown to be important in resolving the work-family conflict in business (Baral and Sampath, 2019) and service relationships (Hennig-Thurau et al., 2006). It helps leaders to influence at different organizational levels (Tee, 2015) and politicians to persuade their voters (Sullivan, 1996; Gabriel and Masch, 2017). And certainly, it is impossible not to mention the fundamental role of contagion in creation and perception of art (Koelsch et al., 2006; Fritz and Koelsch, 2008).

Emotional contagion is thought to be based on two mechanisms: emotional mimicry and afferent feedback (Hatfield et al., 1994). The process of mimicry as copying of emotional expressions of others not only provides physiological reactions in the receiver of emotional contagion, but leads to a subjective experience of the emotions being transferred. This means that emotional mimicry is a crucial mechanism of empathy. However, researchers distinguish emotional contagion from empathy. They emphasize an automatic and involuntary nature of the first one, which in contrast to empathy is not based on elaborate cognitive processes (Hatfield et al., 1992; Doherty, 1997; Decety and Svetlova, 2012). Neurobiologically speaking, the mirror neuron system could underlie the mechanism of emotional contagion (Eren, 2009; Haeusser, 2012), because these neurons are activated during both action and the perception of others’ actions (Rizzolatti et al., 1996).

It is possible to assess the impact of emotional contagion considering changes in a person’s behavior and facial expressions, measuring physiological reactions, such as skin conductance response, as well as using neuroimaging techniques that map neural activity in the brain areas, associated with emotion processing (see Herrando and Constantinides, 2021, for an overview). However, those options are suitable for the assessment of the changes in emotional states and the strength of reaction to the emotional impact at the moment. To measure individual differences in the ability to mimic and synchronize with other people’s emotions, a self-report scale was proposed.

## The Emotional Contagion Scale

Not many attempts to develop a short but reliable scale as an instrument that would capture susceptibility to emotional contagion were made. Thus, Doherty constructed a 38-item version of the scale, which was later downsized to an 18-item questionnaire (Doherty et al., 1995), and finally to a 15-item Emotional Contagion Scale (ECS; Doherty, 1997), which became widely used in psychology and contiguous disciplines.

The ECS showed high reliability (Cronbach’s α = 0.90) and construct validity. The tendency to “catch” emotions of others was positively associated with general emotionality, sensitivity to others and with emotional mode of empathy in contrast to the cognitive one. On the other hand, the ECS was negatively associated with emotional stability (Doherty, 1997; Wróbel and Lundqvist, 2014). Interestingly, the ECS elicited the gender differences in the level of susceptibility to emotional contagion. Thus, women generally defined themselves as more susceptible than men (Doherty, 1997; Lundqvist, 2006; Kevrekidis et al., 2008; Wróbel and Lundqvist, 2014).

The ECS is based on five discrete emotions: happiness, love, fear, anger, and sadness; with three items related to each emotion. The author of the original scale suggested the unidimensional character of the scale as separate factors showed lower reliability than the unidimensional model (Doherty, 1997). The authors of the later adaptations of the questionnaire confirmed the advantages of the unitary application of the ECS in contrast to the five-dimensional (separated discrete emotions) and two-dimensional (positive/negative subscales) options (Lundqvist, 2006; Kevrekidis et al., 2008; Wróbel and Lundqvist, 2014). Therefore, although the multidimensional models of the scale can still be applicable; it is preferable to use the ECS as a unidimensional scale as the reliability of a unified-factor model is more robust.

The ECS was translated into different languages and was adapted and examined on various samples including different cultural contexts: Swedish (Lundqvist, 2006), Brazilian (Gouveia et al., 2007), Japanese (Kimura et al., 2007), Greek (Kevrekidis et al., 2008), Portuguese (Rueff-Lopes and Caetano, 2012), Chinese (Wang et al., 2013), Italian (Lo Coco et al., 2014), Polish (Wróbel and Lundqvist, 2014), and different demographic samples: students (Lundqvist, 2006; Kevrekidis et al., 2008; Wróbel and Lundqvist, 2014), young adults (Lo Coco et al., 2014), adults (Rueff-Lopes and Caetano, 2012). The adaptations showed high reliability and validity of the scale, confirming the possibility to apply the ECS to various subject samples. However, to date, there is no similar instrument to measure individual susceptibility to emotional contagion in the Russian language. Therefore, the aim of this study was to conduct the psychometric analysis of the Russian version of the ECS.

## Materials and Methods

The 15-item ECS questionnaire (Doherty, 1997) was parallel-back translated from English into Russian. We translated original items into Russian language with cultural corrections. Then the translated items were shown to a translator not familiar with the scale and she translated the items back into English. The original English version then was compared to the English translation. Small differences in wording were corrected in preference to the original source. Both the structure and the meaning of the questions were retained as they were in the original scale. The adapted version of the ECS maintained the representation of five discrete emotions: happiness, love, fear, anger, and sadness.

To test the convergent validity, we followed the Portuguese validation of the ECS and chose the Interpersonal Reactivity Index (IRI; Davis, 1980). Twenty-eight items are supposed to measure such empathic facets as fantasy, empathic concern, personal distress, perspective taking (e.g., “I am often quite touched by things that I see happen”). Previously, Neves et al. (2018) found a positive correlation between IRI and ECS.

We also used a newer scale, the Empathy Quotient (EQ; Baron-Cohen and Wheelwright, 2004). It consists of emotional, cognitive and social skill subscales. The EQ consists of 60 items (e.g., “I get upset if I see people suffering on news programs.”), 20 of them being fillers. Botan et al. (2018) showed a positive correlation between the ECS and EQ (both for the total scale and the emotional component).

As for discriminant validity, we did not agree with Rueff-Lopes and Caetano (2012) who used the Perceived stress scale (Cohen et al., 1983) for this purpose. At the same time, they admit that there is a relation between emotional contagion and stress (Omdahl and O’Donnell, 1999). We chose the Sensation Seeking Scale (Zuckerman, 1979), which has not been correlated to the ECS yet, to our knowledge. It demonstrates differences in individual susceptibility toward seeking and undergoing intense sensory experiences. The version we applied (Egorova and Pyankova, 1992) consists of 16 binary items describing risky and safe deeds, from which participants should select one (e.g., “I would prefer to live in an ideal society where everyone is safe, reliable and happy/I would prefer to live in the uncertain, vague days of our history”).

### Participants

A total number of 518 participants, including 319 females (62%) with the mean age 26.67 years (*SD* = 6.59) and 199 men with the mean age 27.08 years (*SD* = 6.43 years) participated in the study by completing the scales online. The mean age of men and women did not differ, *t*(516) = 0.70, *p* = 0.49. All the participants reported no history of neurological or psychological diseases and all of them were native Russian speakers. Educational level of the participants varied from full secondary education to master’s degree.

To measure test–retest reliability, we asked *via* e-mail all the participants to complete the questionnaire once again after 1 month. We received these data from 88 participants (73 females, mean age = 27.8) after 1–3 months from the first participation. For the convergent validity measures, 73 participants (55 females, mean age = 24.6, Cronbach’s α = 0.82 in our study) filled in the IRI (Davis, 1980; Budagovskaja et al., 2013) and 58 participants (51 females, mean age = 26.7, Cronbach’s α = 0.85 in our study) completed the EQ (Baron-Cohen and Wheelwright, 2004; Kosonogov, 2014). As for divergent validity, 101 participants (70 females, mean age = 25.9, Cronbach’s α = 0.70 in our study) filled in the Sensation Seeking scale (Zuckerman, 1979; Egorova and Pyankova, 1992). Like in a study of Rueff-Lopes and Caetano (2012), all these subsamples consisted of different participants. In other words, for example, participants who completed the IRI did not complete the EQ.

### Data Analysis

Kolmogorov–Smirnov tests were applied to test the normality of distributions. Internal reliability was measured as Cronbach’s α and with the split-half method. Gender differences were calculated with *t*-tests for independent samples with Cohen’s *d* as effect size measures. Kaiser–Meyer–Olkin and Bartlett’s tests were performed to confirm the adequacy of the data for factor analysis. Test–retest reliability and external validity were studied with the Pearson correlation analysis. We tested several models of the ECS. First, we applied these measures to a unidimensional model where all 15 items correspond to one factor. Second, we tested the five-factor model with happiness, love, fear, anger, and sadness as factors. Third, following Lundqvist (2006) and Wróbel and Lundqvist (2014), we compiled the two-factor model with fear, anger and sadness in the negative factor and happiness and love in the positive factor.

## Results

### Descriptive Statistics and Reliability

#### One-Factor Model

For the one-factor model we found that the ECS values were normally distributed, Kolmogorov–Smirnov *d* = 0.056, *p* > 0.05, mean = 42.81, *SD* = 6.21, skewness = -0.27, and kurtosis = 0.20. The ECS showed good reliability as a unidimensional scale (Cronbach’s α = 0.78; split-half reliability = 0.83, α_*females*_ = 0.74, and α_*males*_ = 0.78). Test–retest reliability for the whole scale (one-factor model) showed a high value of correlation coefficient, *r*(86) = 0.82, *p* = 0.001. Pairwise comparisons showed the first and second measurement did not differ, *t*(87) = 0.44, *p* = 0.66.

#### Two-Factor Model (Positive/Negative)

In the case of the two-factor model, the values of both subscales were not normally distributed. For the positive subscale, Kolmogorov–Smirnov *d* = 0.11, *p* < 0.01, mean = 19.16, *SD* = 2.89, skewness = -0.72, and kurtosis = 0.80. For the negative subscale, Kolmogorov–Smirnov *d* = 0.15, *p* < 0.05, mean = 23.69, *SD* = 4.44, skewness = -0.19, and kurtosis = -0.07. The reliability was acceptable for both the positive subscale (Cronbach’s α = 0.74; split-half reliability = 0.72) and the negative subscale (Cronbach’s α = 0.72; split-half reliability = 0.72). Test–retest reliability was questionable for the positive subscale [*r*(86) = 0.60, *p* < 0.001, *t*(87) = 1.96, *p* = 0.052] and good for the negative [*r*(86) = 0.82, *p* < 0.001, *t*(87) = 1.62, *p* = 0.11].

#### Five-Factor Model (Love/Happiness/Fear/Anger/Surprise)

For the five-factor model, all the subscales were not normally distributed, Kolmogorov–Smirnov all *d*s > 0.78, all *p*s < 0.01. The reliability was poor (0.51 for sadness, 0.66 for happiness, 0.50 for anger, and 0.51 for fear), except for love (0.70). Test–retest reliability showed acceptable values of correlation coefficient only in the case of sadness [*r*(86) = 0.79, *p* = 0.001, *t*(87) = 1.47, *p* = 0.14] and fear [*r*(86) = 0.76, *p* = 0.001, *t*(87) = 0.87, *p* = 0.39]. For the other subscales, test–retest reliability failed: moderate correlation for happiness [*r*(86) = 0.56, *p* = 0.001, *t*(87) = 1.67, *p* = 0.097] and low correlations for anger [*r*(86) = 0.31, *p* = 0.004, *t*(87) = 8.23, *p* = 0.001] and love [*r*(86) = 0.23, *p* = 0.034, *t*(87) = 8.20, *p* = 0.001].

### Gender Differences

In relation to the gender differences, we found that women were more susceptible to emotional contagion than men, *t*(516) = 8.62, *p* < 0.001, *d* = 0.78, *M*_*females*_ = 44.55, *SD*_*females*_ = 5.47, *M*_*males*_ = 40.03, *SD*_*males*_ = 6.32. The differences were found also both on the negative, *t*(516) = 8.38, *p* < 0.001, *d* = 0.76, *M*_*females*_ = 24.90, *SD*_*females*_ = 3.98, *M*_*males*_ = 21.74, *SD*_*males*_ = 4.46, and on the positive subscales, *t*(516) = 5.55, *p* < 0.001, *d* = 0.50, *M*_*females*_ = 19.70, *SD*_*females*_ = 2.54, *M*_*males*_ = 18.29, *SD*_*males*_ = 3.98. On all five subscales of discrete emotions, women also showed greater values than men, all *t*s > 2.00, all *p*s < 0.046, *d*_*love*_ = 0.18, *d*_*happiness*_ = 0.28, *d*_*fear*_ = 0.52, *d*_*anger*_ = 0.76, *d*_*sadness*_ = 0.79.

#### Correlations Between Subscales

The correlation between the positive and negative subscales was direct and significant, *r* = 0.40, *p* < 0.001. The correlations between all subscales in the five-factor model also were significant (Table 1).

**TABLE 1:** Correlation coefficients between all five subscales of the ECS, all *p*s < 0.002.

	Happiness	Fear	Anger	Sadness
Love	0.21	0.47	0.14	0.23
Happiness		0.31	0.38	0.30
Fear			0.28	0.45
Anger				0.29

## Convergent and Discriminant Validity

The convergent validity was tested with the IRI and EQ questionnaires. The ECS showed low to moderate direct correlations with the overall IRI [*r*(71) = 0.48, *p* < 0.001] and its three subscales: fantasy [*r*(71) = 0.33, *p* = 0.004], empathic concern [*r*(71) = 0.42, *p* < 0.001], and personal distress [*r*(71) = 0.29, *p* = 0.012]. At the same time ECS did not correlate to perspective taking [*r*(71) = 0.19, *p* = 0.100]. Surprisingly, the ECS correlated neither to the overall EQ score, *r*(56) = 0.14, *p* = 0.29, nor its subscale emotional empathy, *r*(56) = 0.04, *p* = 0.76. As to the discriminant validity, the ECS score did not correlate to Sensation Seeking, *r*(99) = -0.01, *p* = 0.92.

### Exploratory Factor Analysis

The measure of sampling adequacy (Kaiser–Meyer–Olkin = 0.81) and Bartlett’s test of sphericity (approximated χ^2^ = 1480, *df* = 105, *p* = 0.001) showed that the data were adequate for a factor analysis procedure. As shown in Table 2, items did not fit the presumed five factors. All three negative factors did not correspond to the theoretically expected items. In other words, items 1, 4, and 14 theoretically should fit to a factor “sadness.” However, the 8 (theoretically describing fear) fit the same factor. The items 7 and 10 should fit to a factor “anger,” but the item 13 (presumably, a fear one) fit this factor as well. At the same time, the items 5 (an anger one) and 15 (a fear one) comprised another factor.

**TABLE 2:** Factor loadings of the ECS items.

	Factor – 1	Factor – 2	Factor – 3	Factor – 4	Factor – 5
1 Sadness	–0.03	0.02	**0.65**	0.13	0.26
2 Happiness	0.00	0.10	0.10	**0.81**	0.04
3 Happiness	0.05	0.35	0.19	**0.51**	0.09
4 Sadness	0.11	0.06	**0.71**	0.25	–0.03
5 Anger	0.13	–0.01	0.06	0.17	**0.79**
6 Love	–0.02	**0.71**	–0.03	0.20	0.21
7 Anger	**0.74**	0.10	–0.15	0.03	0.19
8 Fear	0.23	0.01	**0.53**	0.16	0.23
9 Love	0.15	**0.78**	0.16	0.07	–0.08
10 Anger	**0.78**	0.11	0.22	–0.02	0.01
11 Happiness	0.15	0.23	0.09	**0.74**	0.12
12 Love	0.14	**0.77**	0.01	0.17	0.04
13 Fear	**0.70**	0.09	0.16	0.21	0.17
14 Sadness	0.17	0.31	**0.53**	–0.25	0.22
15 Fear	0.17	0.15	0.22	–0.04	**0.69**
**Explained variance**	**1.85**	**2.03**	**1.72**	**1.78**	**1.41**
**Proportion of total**	**0.12**	**0.14**	**0.11**	**0.12**	**0.09**

*Loadings higher than 0.40 are written in bold.*

We also followed the idea of Kevrekidis et al. (2008) and deleted three items related to fear. This helped us to obtain a four-factor model, in which all 12 items fit the four factors (anger, sadness, happiness, and love), theoretically proposed by Doherty (Supplementary Table 1).

Then we tested the two-factor model (Table 3). In other words, we subjected the 15 items of the ECS to the factor analysis with the maximum of two factors. All the 15 items fit well into the model. However, the proportion of explained variance was lower than in the unidimensional model.

**TABLE 3:** Factor loadings of the ECS items along two factors.

	Factor – 1 (negative)	Factor – 2 (positive)
1 Sadness	**0.46**	0.16
2 Happiness	0.04	**0.61**
3 Happiness	0.16	**0.61**
4 Sadness	**0.43**	0.26
5 Anger	**0.52**	0.11
6 Love	0.06	**0.66**
7 Anger	**0.50**	0.07
8 Fear	**0.55**	0.15
9 Love	0.13	**0.64**
10 Anger	**0.64**	0.08
11 Happiness	0.17	**0.66**
12 Love	0.10	**0.69**
13 Fear	**0.63**	0.21
14 Sadness	**0.52**	0.11
15 Fear	**0.59**	0.10
**Explained variance**	**2.73**	**2.72**
**Proportion of total**	**0.18**	**0.18**

*Loadings higher than 0.40 are written in bold.*

### Confirmatory Factor Analysis

Finally, we conducted a confirmatory factor analysis for several models, discussed in the literature (Table 4). χ^2^/*df* was higher than 2 only for the one-factor model. RMSEA, SRMR, FGI, AGFI, CFI, and NFI values were good for all models.

**TABLE 4:** Confirmatory factor analysis for different models of the ECS.

model	χ ^2^	*df*	*p*	χ ^2^/*df*	RMSEA	SRMR	GFI	AGFI	CFI	NFI
One-factor	97.2	48	0.001	2.03	0.044	0.034	0.976	0.939	0.965	0.922
Two-factor (pos/neg)	114.5	61	0.001	1.87	0.040	0.038	0.972	0.945	0.962	0.923
Four-factor (l/h/a/s)	61.8	33	0.002	1.87	0.041	0.032	0.980	0.954	0.971	0.942
Five-factor (l/h/f/a/s)	84.4	58	0.013	1.45	0.029	0.030	0.979	0.957	0.981	0.944

## Discussion

The aim of the work was to examine the ECS in a Russian sample. The adaptation of the ECS showed normal distribution, good internal and test–retest reliability in the case of a unidimensional scale, which represents all five emotions: happiness, love, fear, anger, and sadness together. The results on internal consistency (α = 0.78), are lower than in the original scale (Doherty, 1997), as well as slightly lower than in some validation studies (Brazilian: Gouveia et al., 2007; Portuguese: Rueff-Lopes and Caetano, 2012; Polish: Wróbel and Lundqvist, 2014). However, they are little better than the results of other adaptations (Swedish: Lundqvist, 2006; Greek: Kevrekidis et al., 2008). Such results demonstrate the acceptability of using the full ECS as a measurement of predisposition toward emotional contagion in different cultural contexts.

As we expected, the test of discriminant validity showed no correlation of emotional contagion to sensation seeking. As for convergent validity, the ECS correlated to the IRI and its subscales (except for perspective taking). In the Portuguese adaptation (Rueff-Lopes and Caetano, 2012), however, only the total IRI was used and it did correlate to the ECS. In a study of Neves et al. (2018), the IRI and ECS also positively correlated. Contrary to our expectations, the ECS did not correlate to the EQ and its emotional component. We could find only one work, in which both the ECS and EQ were measured (Botan et al., 2018). The ECS showed a direct correlation to the emotional component of the EQ (*r* = 0.49), yet it was lower between the ECS and the total EQ (*r* = 0.33). As for the relationship between the ECS and IRI, there were low, but significant, correlations between the ECS and the total IRI (*r* = 0.18), empathic concern (*r* = 0.20) and personal distress (*r* = 0.20), but not perspective taking and fantasy (both *r*s < 0.07; all the values calculated by us, thanks to the open dataset). Therefore, further investigations with specific designs and larger samples are needed to clarify the relationship between emotional contagion and empathy. Worth to note, that, contrary to the ECS, the empathy questionnaires did not contained discrete emotion subscales. We consider fruitful future possible investigations of the relation between contagion and empathy over discrete emotions. For this purpose, one could use such methods as empathic anger scale (Vitaglione and Barnett, 2003) or an empathy questionnaire after a discrete emotion induction (Xu et al., 2019).

The division of the scale into the two-factor model with the positive (happiness, love) and negative (fear, anger, and sadness) affects demonstrated acceptable reliability for each subscale. Such results are congruent with the Brazilian, Greek, and Polish adaptations of the ECS (Gouveia et al., 2007; Kevrekidis et al., 2008; Wróbel and Lundqvist, 2014), albeit even better as both negative and positive factors showed acceptable Cronbach’s α. The test–retest reliability in the two-factor model was acceptable only for the negative subscale. However, both of them showed non-normal distribution with the negative skewness, that is the mean values were greater than the middle of the scale. In other words, people are more contagious than the middle value (which would be 12 for positive subscale and 18 for the negative one). Overall, this means that the Russian adaptation of the ECS can be applicable, with a great caution, to measure individual differences in emotional contagion for positive and negative emotions separately.

The five-factor model, based on the separation of each of the basic categories presented in the ECS, showed abnormal distribution, poor internal consistency, and not acceptable test–retest reliability for three subscales. Such results are generally consonant with the results of Greek and Polish validation. They can be explained by the small number of items in each factor. Interestingly, other validation studies did not test distribution parameters of five subscales. Presumably, three items are not enough to obtain acceptable psychometric properties of a scale. A possible future direction would be to elaborate a questionnaire, which would contain more questions for each discrete emotion. Otherwise, one could think about specific contagion questionnaires for each discrete emotion.

In the exploratory factor analysis, the negative emotions (anger, fear, and sadness) did not fit into three different factors. The withdrawal of the fear items improved the distribution of other items to four factors (happiness, love, anger, and sadness), which is similar to the results of the Greek adaptation study (Kevrekidis et al., 2008). This may mean that the negative emotion items in the ECS were not clear for the participants, or the same model did not result to be consistent. Therefore, another solution would be to exclude fear questions in the future versions of the emotional contagion questionnaire.

The results of the validation show that the one-factor model – the full ECS scale – had, in overall, better psychometric properties than two- and five-factor models. Susceptibility to emotional contagion might be different in case of different emotions and different valence of emotions. However, we do not recommend applying the 15-items ECS scale as a five-factor model that measures predisposition to emotional contagion for the discrete emotions.

Furthermore, similar to the results of many other adaptations of the ECS (Lundqvist, 2006; Gouveia et al., 2007; Kevrekidis et al., 2008; Rueff-Lopes and Caetano, 2012; Wróbel and Lundqvist, 2014), we found that women were more contagious emotionally than men. Women reported greater emotional contagion values within each model we tested, that is in the overall score, positive and negative subscales, and five emotion scales. This finding may entail that the proportion of men and women should be equalized between groups under study in the experiments related to emotional contagion, emotional physiological reactivity, embodied cognition, and so on.

## Conclusion

After the examination of the ECS within a Russian sample, we confirmed that the questionnaire is a reliable instrument of measurement of the individual predisposition to emotional contagion and it can be used in the Russian population. We recommend only the use of the one-factor version or the two-factor version (positive and negative subscales, but with caution). Future researchers should also take into account gender differences that is women display greater emotional contagion.

## Data Availability Statement

The raw data supporting the conclusions of this article will be made available by the authors, without undue reservation.

## Ethics Statement

The studies involving human participants were reviewed and approved by Ethics Committee of HSE University. The patients/participants provided their written informed consent to participate in this study.

## Author Contributions

Both authors listed have made a substantial, direct, and intellectual contribution to the work, and approved it for publication.

## Conflict of Interest

The authors declare that the research was conducted in the absence of any commercial or financial relationships that could be construed as a potential conflict of interest.

## Publisher’s Note

All claims expressed in this article are solely those of the authors and do not necessarily represent those of their affiliated organizations, or those of the publisher, the editors and the reviewers. Any product that may be evaluated in this article, or claim that may be made by its manufacturer, is not guaranteed or endorsed by the publisher.
