# Supplementary material for: A Russian Adaptation of the Emotional Contagion Scale
Source: Front Psychol. 2022 Aug 17;13:872718. doi: 10.3389/fpsyg.2022.872718 (PMC9431020; doi:10.3389/fpsyg.2022.872718)
Supplement: Supplementary file 1 [file Data_Sheet_1.docx]

S-Figure 1.The distribution of the sample’s results of the ECS based on the level of emotional contagion in relation to the frequency of results


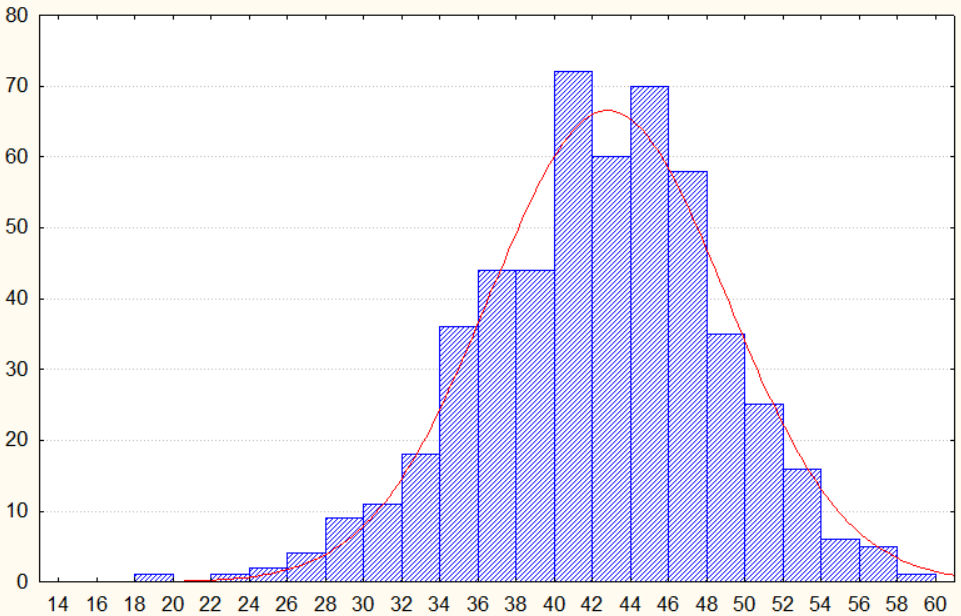


S-Table 1. Factor loadings of the ECS items, beside fear ones.

|  | **Factor - 1 (love)** | **Factor - 2 (anger)** | **Factor - 3 (happiness)** | **Factor - 4 (sadness)** |
| --- | --- | --- | --- | --- |
| ***1 sadness*** | -.02 | -.01 | .18 | **.79** |
| ***2 happiness*** | .09 | -.04 | **.81** | .08 |
| ***3 happiness*** | .34 | .07 | **.53** | .17 |
| ***4 sadness*** | .09 | .04 | .26 | **.62** |
| ***5 anger*** | -.10 | **.43** | .31 | .30 |
| ***6 love*** | **.68** | .02 | .24 | .05 |
| ***7 anger*** | .09 | **.84** | .06 | -.05 |
| ***9 love*** | **.80** | .09 | .06 | .12 |
| ***10 anger*** | .17 | **.72** | -.03 | .20 |
| ***11 happiness*** | .23 | .13 | **.75** | .06 |
| ***12 love*** | **.77** | .14 | .18 | .01 |
| ***14 sadness*** | .27 | .20 | -.19 | **.65** |
| **Explained Variance** | 2.00 | 1.50 | 1.83 | 1.60 |
| **Proportion of total** | .17 | .13 | .15 | .13 |

S-Table 2. Multi-group confirmatory factor analysis for 1- and 2-factor models of the ECS.

| **model** | **χ2** | ***df*** | ***p*** | **χ2*/df*** | **RMSEA** | **SRMR** | **GFI** | **CFI** | **NFI** |
| --- | --- | --- | --- | --- | --- | --- | --- | --- | --- |
| 1-factor |  |  |  |  |  |  |  |  |  |
| configural | 169.5 | 96 | .001 | 1.77 | .054 | .046 | .957 | .942 | .885 |
| metric | 200.0 | 110 | .001 | 1.82 | .055 | .058 | .951 | .933 | .868 |
| scalar | 284.8 | 124 | .001 | 2.30 | .071 | .070 | .992 | .873 | .808 |
| 2-factor (pos/neg) |  |  |  |  |  |  |  |  |  |
| configural | 200.4 | 126 | .001 | 1.59 | .048 | .051 | .951 | .941 | .865 |
| metric | 229.5 | 139 | .001 | 1.65 | .050 | .062 | .944 | .929 | .845 |
| scalar | 326.8 | 152 | .001 | 2.15 | .067 | .071 | .990 | .810 | .779 |

Table S2. Russian Version of the Emotional Contagion Scale

|  | Не согласен - согласен |
| --- | --- |
| 1. Если кто-то, с кем я разговариваю, начинает плакать, у меня наворачиваются слезы на глазах.  2. Нахождение рядом со счастливым человеком поднимает мне настроение, когда я чувствую себя подавленным (подавленной).  3. Когда кто-то тепло улыбается мне, я улыбаюсь в ответ и чувствую тепло внутри.  4. Меня переполняет печаль, когда люди говорят о смерти своих близких.  5. Я сжимаю челюсти, и мои плечи напрягаются, когда я вижу в новостях разгневанные лица.  6. Когда я смотрю в глаза любимого человека, мой разум наполняется мыслями о романтике.  7. Меня раздражает находиться рядом с разгневанными людьми.  8. Глядя на испуганные лица жертв в новостях, я пытаюсь представить, что они могли чувствовать.  9. Я таю, когда тот (та), кого я люблю, прижимает меня к себе.  10. Я напрягаюсь, когда слышу гневную ссору.  11. Когда я нахожусь в окружении счастливых людей, мой разум наполняется счастливыми мыслями.  12. Я ощущаю, как мое тело реагирует, когда любимый человек касается меня.  13. Я замечаю, что сам (сама) становлюсь напряженным (напряженной), когда нахожусь в окружении людей. испытывающих стресс.  14. Я плачу над грустными фильмами.  15. Слушая пронзительные крики перепуганного ребенка в приемной зубного врача, я сам (сама) начинаю нервничать. | 1 2 3 4  1 2 3 4  1 2 3 4  1 2 3 4  1 2 3 4  1 2 3 4  1 2 3 4  1 2 3 4  1 2 3 4  1 2 3 4  1 2 3 4  1 2 3 4  1 2 3 4  1 2 3 4  1 2 3 4 |
| *Ключ*: Радость = 2, 3, 11; Любовь = 6, 9, 12; Страх = 8, 13, 15; Гнев = 5, 7, 10; Печаль = 1, 4, 14. Все вопросы прямые |  |
